# Evolution from Decompressive Craniectomy to Early Minimally Invasive Surgical Approach for Refractory Increased Intracranial Pressure Treatment: Merit or Social Problems?

**DOI:** 10.4274/TJAR.2024.241696

**Published:** 2025-07-24

**Authors:** Rudin Domi, Filadelfo Coniglione, Gentian Huti, Mario Dauri, Asead Abdyli, Krenar Lilaj, Federico Bilotta

**Affiliations:** 1 University of Medicine, Tirana (UMT), Department Anaesthesiology and Intensive Care, Tirana, Albania; 2 University of Rome Tor Vergata, Department of Anaesthesia and Intensive Care Medicine, Rome, Italy; 3 Sapienza University of Rome, Department of Anaesthesiology and Intensive Care, Rome, Italy

**Keywords:** Decompressive craniectomy, intensive care unit, intracranial pressure, ischemic stroke, traumatic brain injury

## Abstract

In conclusion, treating increased intracranial pressure is a significant challenge for physicians in intensive care units and emergency departments. If not managed properly, elevated intracranial pressure can lead to brain edema, reduced oxygenation, and, ultimately, death. Intracranial hypertension can be caused by various conditions, including traumatic brain injury, massive intracranial bleeding, and large ischemic stroke, such as middle cerebral artery thrombosis. Treatment consists of both pharmacological and surgical. Surgical treatments include early surgical evacuation and decompressive craniectomy (DC). DC is a critical intervention for managing refractory intracranial hypertension when all conventional therapies fail. It is a decisive step that is intended to save lives and minimize long-term neurological deficits. The procedure must be carefully planned and executed based on the patient’s specific clinical scenario and needs. The decision to proceed with DC should be based on a comprehensive assessment of the patient’s condition, the effectiveness of other treatments, and the potential benefits and risks of the procedure. If all conventional pharmacological and non-pharmacological therapies fail and intracranial hypertension persists, regardless of the underlying cause, DC is indicated and can be considered a critical intervention. Currently, surgical treatment has gained popularity, and many papers have been published. This review summarizes the tendencies in the literature.

**Main Points**

• Surgical treatment can effectively reduce refractory malignant intracranial pressure.

• The functional outcome of decompressive craniectomy remains uncertain although it appears to be generally poor.

• The prognosis for patients is influenced by the cause and severity of increased intracranial pressure.

## Introduction

Decompressive craniectomy (DC) is usually performed when standard medical therapy fails to maintain intracranial pressure (ICP) below 20 mmHg. The procedure helps reduce the increased ICP, thus increasing cerebral blood flow and cerebral oxygenation. Although skull decompression has been reported both before and after the medieval period, the first documented decompressive surgery in modern times was performed by Marcotte.^1^ Kocher^2^ was the first to report the use of DC after traumatic brain injury (TBI). After Kocher,^2^ Cushing^3^ expanded the indications for DC. He reported performing the procedure to address increased ICP resulting from brain tumors and penetrating brain injuries. Cushing’s^4^ work significantly influenced the broader acceptance and utilization of DC in neurosurgery. In the mid-1970s, there were significant reports and published papers by various authors detailing the use and outcomes of DC. These publications highlighted serious cases in which DC was employed, contributing to the growing body of evidence supporting the procedure’s effectiveness in managing increased ICP due to various causes, such as TBIs and other conditions.^5,6^ Nowadays, DC is a standardized procedure for managing severe, refractory increased ICP. Numerous studies and published papers support the efficacy of DC in improving outcomes in patients with conditions such as TBI, stroke, and other causes of elevated ICP. These studies have provided robust evidence demonstrating that DC can significantly reduce mortality and improve functional outcomes in appropriately selected patients.^7,8^ DC is typically reserved for cases in which third-tier therapies are unsuccessful. By reducing ICP, DC can significantly decrease mortality. Several situations justify the use of DC, including TBI, subdural hematoma, and cerebrovascular diseases, such as: hemispheric ischemia and hemorrhage- induced intracranial hypertension. These conditions can lead to critically elevated ICP, where standard medical treatments fail to provide adequate relief. In such scenarios, DC becomes a crucial intervention to mitigate the risk of severe brain damage and improve patient outcomes.^9,10^

TBI is a significant public health concern and a major cause of increased ICP, contributing to global mortality rates. TBI can be caused by either blunt or penetrating trauma to the head. It is associated with a wide range of physiological and organic brain damage, leading to various degrees of brain malfunction.

The physiological consequences of TBI include disrupted blood flow, swelling, and increased ICP, which can further damage brain tissue and impair its function. Organic damage can involve bleeding (hematomas), bruising, and neuronal death. The combination of these factors can lead to a wide range of symptoms, from mild cognitive impairment to severe disability or even death.

Effective management of TBI involves prompt medical assessment, imaging studies like computed tomography or magnetic resonance imaging scans, and interventions to stabilize the patient and minimize secondary brain injury. Treatment strategies may include surgical procedures to relieve pressure, medications to control symptoms, and rehabilitation to support recovery and improve long-term outcomes.^11^ Increased ICP and cerebral edema are the main complications of TBI. Due to these phenomena, further cerebral ischemia and brain herniation can occur, making DC an alternative to conventional medical therapy in patients with intracranial hypertension.

### Pathophysiology of Increased ICP and Its Treatment

The Monro-Kellie doctrine explains the dynamics of the three main components within the cranial vault: brain tissue, cerebral blood flow, and cerebrospinal fluid (CSF). According to this doctrine, the cranial compartment is incompressible, and the volume inside the cranium is fixed. Therefore, an increase in the volume of any one of the intracranial constituents must be compensated by a decrease in the volume of another; otherwise, ICP will increase. Brain tissues comprise most of the intracranial content. If there is an increase in brain tissue volume, such as from swelling or a mass lesion, compensatory mechanisms should be initiated. Cerebral blood flow and volume can change rapidly to compensate for ICP changes. For example, vasoconstriction or vasodilation of cerebral blood vessels can occur in ICP management. CSF can be displaced into the spinal canal, its production can be decreased, and absorption can be increased to balance the ICP. When one of these elements increases, the body attempts to maintain a normal ICP by decreasing the volume of one or both components. However, these compensatory mechanisms are limited. If compensatory mechanisms are overwhelmed, ICP can increase, causing severe neurological damage or death if not managed promptly.^12^ Therefore, if brain content is increased by tumors, edema, and bleeding, the other components (cerebral blood flow and CSF) tend to be diminished to maintain a normal ICP value. When compensatory mechanisms are exhausted, ICP can increase, leading to intracranial hypertension. The normal ICP value is typically 5-15 mmHg. Intracranial hypertension is defined as an ICP>22 mmHg sustained for at least 5 minutes. Initially, the body attempts to compensate for increased intracranial volume (e.g., from edema, mass lesions, or hemorrhage) by displacing CSF into the spinal canal and reducing cerebral blood volume. However, these mechanisms have limitations. As ICP continues to rise beyond compensatory limits, the brain attempts to maintain homeostasis by directing blood (particularly venous blood) and CSF out of the skull. Increased ICP can cause brain tissue to swell (edema) and shift from a normal midline position. This shift can further impair cerebral vascularization, particularly affecting blood flow to critical brain areas. As ICP rises, blood vessels are compressed, thereby reducing cerebral blood flow. This leads to decreased oxygen and nutrient delivery to brain tissue, causing cerebral ischemia. Reduced blood flow and oxygenation exacerbate brain tissue damage, leading to a vicious cycle in which ischemia causes more swelling (edema), which in turn raises ICP further. As cerebral tissue becomes increasingly deprived of oxygen, it swells, further increasing the ICP. This condition creates a feedback loop of worsening edema and ICP. Ultimately, if not managed, this can lead to severe consequences, including brain tissue herniation, significant neurological impairment, and potentially death. Managing elevated ICP often involves medical interventions to reduce brain swelling, optimize cerebral blood flow, and sometimes surgical procedures to remove mass lesions or CSF to lower pressure. Prompt recognition and treatment of elevated ICP are crucial to prevent irreversible brain damage.^13^ Finally, increased ICP diminishes cerebral oxygenation, forces brain to “escape” in anatomic holes i.e. brain herniation. The final stage is brain death.

Many publications have focused on increased ICP treatment, particularly after TBI and intracranial hemorrhage. After the exclusion of neurosurgical emergency, medical therapy is initiated to manage the increased ICP. Standard early treatment begins after a carefully neurological examination. Glasgow Coma Scale (GCS) score of 8 points indicates the patient intubated and sedated. The recommended head position is 30 grades upright and neutral to avoid any situations that can impair venous return. The patient needs to be fully sedated, and the use of muscle relaxants is crucial to avoid any increase in ICP during coughing and spontaneous forced respiration. Osmotic therapy using mannitol and hypertonic saline. It is of great importance to avoid hypotension and to maintain blood pressure above 110 mmHg. Hyperventilation is reserved only in cases of sudden increased ICP, maintaining a PaCO_2_ around 30-35 and taking into consideration that aggressive CO_2_ reduction can lead to vasoconstriction and further ischemia. The aggressive treatment of fever and seizures is crucial to prevent secondary brain damage. Barbituric coma is used in severe TBI with refractory uncontrollable increased ICP.^14^ There are no reports of the efficacy of hypothermia in patients with TBI and intracranial hypertension.^15^ The three-tier therapy model includes all therapeutics in three tiers depending on severity.^16,17^ Tier Zero includes early evaluation and admission (neurological evaluation, intubation, ventilation, monitoring, and sedation); Tier One is focused on cerebral perfusion pressure (CPP) 60-70 mmHg, analgesia and sedation, osmolar therapy, and antiseizures treatment; Tier Two is composed by the combination of mild hypocapnia (PaCO_2_ 30-35 mmHg), neuromuscular blocking, and mean arterial pressure adjusting according to adequate CPP; Tier Three includes barbituric coma, mild hypothermia (32-35 grades Celsius), and DC.^18^ Robba et al.^19^ published data from their study SYNAPSE-ICU. The aim of this study was to provide a full picture of the increased use of ICP treatment modalities and differences among countries and institutions. This study enrolled 2,320 patients with the following inclusion criteria: patients aged ≥18 years, traumatic injury/ICP/subarachnoid hemorrhage, GCS score 7 (E1, V1, M≤5), or new neurological deterioration in intensive care unit (ICU) within 48 hours after ICU admission. They found that therapies to control increased ICP are generally used, and aggressive treatment modalities seem to have a positive effect on 6-month mortality.

If all conventional pharmacological and non- pharmacological therapies fail and intracranial hypertension persists independently as the cause of increased ICP, DC is indicated and can be considered.^20^

### Decompressive Craniectomy

Prior to performing DC, patients must exclude the non- efficacy of other treatments and the indication for this procedure. DC helps increase cerebral blood flow, reduce damage size, and reduce ICP. Surgical plan and extension depend on damage location, size of damage, bilateral or unilateral ICP value, and presence of external ventricular drainage. DC may be performed as hemi-craniectomy or bilateral craniectomy.^20,21^ In hemispheric craniectomy, a larger frontoparietal skull fragment (up to 15 cm) is performed. Several early and late complications have been reported.^22^ These complications include wound infections, meningitis, CSF leakage, external herniation, bleeding, postoperative seizures, subdural hygroma, and hydrocephalus.^22^ The indications for DC include TBI, malignant hemispheric ischemia (middle cerebral artery thrombosis), intracranial hemorrhage, intracranial infection, massive brain tumors, and cerebral venous system thrombosis.^23^ DC can control increased ICP, but the prognosis and their permanent disabilities are not known. Therefore, appropriate indication, careful patient selection, a detailed surgical plan, and detailed explanations for relatives are of great importance.

### Early Surgical Treatment and DC in Different Clinical Scenarios

Hutchinson et al.^24^ conducted Randomized Evaluation of Surgery with Craniectomy Evacuation for Acute Subdural Hematoma-*RESCUE-ASDH *trial. The study included patients with acute traumatic subdural hematoma scheduled for craniotomy or DC.^24^ The patients were divided into 2 groups: 228 patients who underwent craniotomy and 222 patients who underwent DC. The authors measured as endo-points: outcome measured by Glasgow Outcome Scale-Extended (GOSE) at 12 months after surgery. They reported that the rates of serious disability and quality of life did not statistically significantly changes in compared groups.^24,25^ DCs were primarily used for high-refractory ICP treatment after TBI. Several authors reported their data when DC regained popularity, emphasizing the crucial role of DC in controlling intracranial hypertension.^26,27^ The randomized evaluation of surgery with craniectomy for uncontrollable elevation of intracranial pressure (RESCUEicp) and decompressive craniectomy in patients with severe traumatic brain injury (DECRA) trials remain the main trials for DC after TBI intracranial hypertension.

The RESCUEicp trial was published in 2016, and the study aimed to evaluate the efficacy of DC in controlling ICP.^28^ The authors’ endpoint was the 6-month evaluation of the GOSE (8 grades from “death” to “good recovery”) at 6 months after. The multicenter study included 408 patients suffering from ICP>25 mmHg for 1-12 hours. Interesting conclusions came from the study, establishing DC as an effective method to control increased ICP, but a high percentage of vegetative state in survivals. DECRA trial conclusions were recently published, arriving in the same conclusions as RESCUEicp.^29,30^ Kolias et al.^31^ published an interesting paper in JAMA 2022, expanding the GOSE up to 24 months after treatment. They enrolled 408 patients (206 in the surgical group and 202 in the medical group). Interesting data emerged from the study, revealing that even extended RESCUEicp produced the same findings as RESCUEicp, but the surgical group showed more improvement over months compared with the medical group.^31^

Intracranial bleeding is a major cause of ICU admission. Different papers have been published, and several data have been reported in the literature. Mendelow et al.^32^ published in Lancet their first study called surgical trial in intracerebral hemorrhage (STICH). They enrolled 1033 patients from 87 centers, and the endpoint was the GOSE at 6-month follow-up. Mendelow et al.^33^ found no difference between early surgery and medical treatment between groups. The STICH II trial results were published in 2013.^33^ The authors compared early surgery with medical treatment in patients with superficial lobar intracerebral bleeding without intraventricular hemorrhage. The results found no significance in rate deaths and slight clinical improvement in patients who underwent early surgical treatment. The minimally invasive surgery with thrombolysis in intracerebral haemorrhage evacuation phase III (MISTIE III) study was a randomized and controlled trial.^34^ The authors included 506 patients divided into the MISTIE and standard medical groups. The MISTIE III group concluded that reduction in the size of bleeding was associated with improved prognosis. Pradilla et al.,^35^ in their published paper in the New England Journal of Medicine, reported data on early minimally invasive removal of intracerebral hemorrhage. This multicenter randomized trial enrolled 300 patients with basal ganglia bleeding and lobar hemorrhage. The 30-day mortality rates were 9.3% in the surgery group and 18.0% in the control group. The 180-day functional outcomes were significantly better in the group with early invasive evacuation of hematoma.

## Conclusion

DC and other early surgical approaches decrease ICP and improve patients outcome. The long-term effects of functional outcomes are prone of controversies and not yet clear. The surgical approach must be strongly considered as an effective option to treat increased ICP, even when associated with unclear benefits for functional outcomes.
